# Characterization of the complete chloroplast genome of *Phleum pratense* L. cv. Minshan

**DOI:** 10.1080/23802359.2019.1692706

**Published:** 2019-11-21

**Authors:** Guangxin Cui, Yuan Lu, Xiaoxing Wei, Xiaoli Wang, Chunmei Wang, Yaqin Gao, Huirong Duan

**Affiliations:** a Lanzhou Institute of Husbandry and Pharmaceutical Science, Chinese Academy of Agricultural Sciences, Lanzhou, Gansu, China;; b Academy of Animal and veterinary sciences, Qinghai University, Xining, Qinghai, China;; c Laboratory of Quality & Safety Risk Assessment for Livestock Products, Ministry of Agriculture and Rural Affairs, Lanzhou Institute of Husbandry and Pharmaceutical Science, Chinese Academy of Agricultural Sciences, Lanzhou, Gansu, China

**Keywords:** *Phleum pratense* L. cv. Minshan, chloroplast genome, phylogenetic relationship

## Abstract

Severe seed degradation of *Phleum pratense* L. cv. Minshan restricts its productivity and promotion, the chloroplast genome and evolutionary relationship analysis of Minshan could provide inheritance reasons on seed degradation and fundamental genetic reference for its molecular breeding and biological research. Its chloroplast genome was 134,973 bp in length, containing a pair of inverted repeated regions (42,726 bp) which were separated by a large single copy region of 79,473 bp, and a small single copy region of 12,774 bp. Moreover, a total of 114 functional genes were annotated, including 79 mRNA, 32 tRNA genes, and 5 rRNA genes. The phylogenetic relationships of 25 species indicated that Minshan was closely related to *Avena damascene*.

*Phleum pratense* L. cv. Minshan is the first and the only one cultivar of China, approved by the Chinese Herbage Cultivar Registration Board in 1990, which is highly recommended for horses due to the rich source of fiber, high yield and environmental adaptability to Minshan region (Cao 2003). In the last 20 years, the development of Minshan has dissipated into a wild reproduction state due to poor market demand and the lag of seed breeding (Du 2003). Seed purification and rejuvenation and new cultivars suitable to local environment are strongly demanded to meet the requirements of developing horsing industry in recent years (Wang et al. 2018). To provide new insights on seed degradation and fundamental genetic reference for future biological research and molecular breeding programs of *P. pratense*, the genetic diversity of Minshan was reported in this study. The complete chloroplast genome sequence of Minshan based on Illumina NovaSeq platform was determined (Genbank accession number: MN551180) to provide valuable complete chloroplast genomic information and the phylogeny of Minshan was illustrated based on the complete chloroplast genome sequences as well.

The fresh leave samples of Minshan were collected in Lanzhou Scientific Observation and Experiment Field Station of the Ministry of Agriculture for Ecological System in the Loess Plateau Area (36re co, 103re collected in 1700 m), Gansu, China, on 22 July 2019. The voucher specimen was kept in Herbarium of Lanzhou Institute of Husbandry and Pharmaceutical Science, Chinese Academy of Agricultural Sciences (CYSLS-PpMSCUI20190722). Total genomic DNA extraction and genome sequence assembling were conducted by Benagen Tech Solution Co., Ltd (Wuhan, China). The assembled genome was annotated using GeSeq (Tillich et al. 2017).

The complete cp genome of Minshan was 134,973 bp in length with a typical quadripartite structure, containing a pair of inverted repeated (IR) regions (42,726 bp) that are separated by a large single copy (LSC) region of 79,473 bp, and a small single copy (SSC) region of 12,774 bp. The GC content of the whole cp genome was 38.43%. A total of 114 functional genes were annotated, including 79 protein-coding genes (mRNA), 32 tRNA genes, and 5 rRNA genes. The protein-coding genes, tRNA genes, and rRNA genes account for 69.30, 28.07, and 4.39% of all annotated genes, respectively.

Phylogenetic analysis was completed on an alignment of concatenated nucleotide sequences of all complete chloroplast genomes of Minshan and other 24 species (Figure 1). MAFFT (Katoh and Standley 2013) was used to conduct alignment and the neighbor-joining (NJ) method was employed to build a phylogenetic tree with bootstrap set to 1000. The results showed that Minshan had a closer relationship with *Avena damascena*.

**Figure 1.**
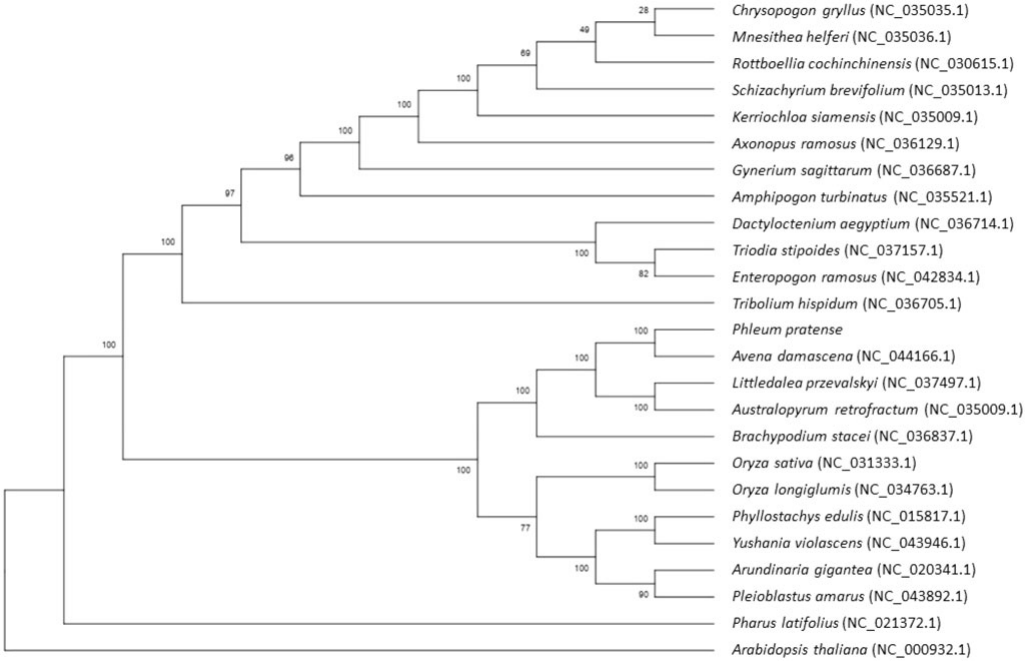
Phylogenetic relationships of 25 species based on complete chloroplast genome using the neighbor-joining methods. The bootstrap values were based on 1000 replicates and are shown next to the branches.

This study provided the complete chloroplast genome structure traits of *P. pratense* L. cv. Minshan and showed the phylogenetic relationships with other 24 species. This information will contribute to its future breeding and biological research and find reasons for seed degradation.
